# Residential Radon Exposure and Skin Cancer Incidence in a Prospective Danish Cohort

**DOI:** 10.1371/journal.pone.0135642

**Published:** 2015-08-14

**Authors:** Elvira Vaclavik Bräuner, Steffen Loft, Mette Sørensen, Allan Jensen, Claus Erik Andersen, Kaare Ulbak, Ole Hertel, Camilla Pedersen, Anne Tjønneland, Susanne Krüger Kjær, Ole Raaschou-Nielsen

**Affiliations:** 1 Research Center of Prevention and Heath, Center of Health, Capital region of Denmark, Glostrup Hospital, Glostrup, Denmark; 2 Diet, Genes and Environment, Danish Cancer Society Research Centre, Copenhagen, Denmark; 3 Department Public Health, Section of Environmental Health, Faculty of Health Sciences, University of Copenhagen, Copenhagen, Denmark; 4 Virus, Lifestyle and Genes, Danish Cancer Society Research Centre, Copenhagen, Denmark; 5 Center for Nuclear Technologies, Technical University of Denmark, Roskilde, Denmark; 6 National Institute of Radiation Protection, Herlev, Denmark; 7 Department of Gynecology, Rigshospitalet, University of Copenhagen, Copenhagen, Denmark; 8 Department of Environmental Science, Aarhus University, Aarhus, Denmark; Ohio State University Medical Center, UNITED STATES

## Abstract

**Background:**

Although exposure to UV radiation is the major risk factor for skin cancer, theoretical models suggest that radon exposure can contribute to risk, and this is supported by ecological studies. We sought to confirm or refute an association between long-term exposure to residential radon and the risk for malignant melanoma (MM) and non-melanoma skin cancer (NMSC) using a prospective cohort design and long-term residential radon exposure.

**Methods:**

During 1993–1997, we recruited 57,053 Danish persons and collected baseline information. We traced and geocoded all residential addresses of the cohort members and calculated radon concentrations at each address lived in from 1 January 1971 until censor date. Cox proportional hazards models were used to estimate incidence rate-ratios (IRR) and confidence intervals (CI) for the risk associated with radon exposure for NMSC and MM, and effect modification was assessed.

**Results:**

Over a mean follow-up of 13.6 years of 51,445 subjects, there were 3,243 cases of basal cell carcinoma (BCC), 317 cases of squamous cell carcinoma (SCC) and 329 cases of MM. The adjusted IRRs per 100 Bq/m^3^ increase in residential radon levels for BCC, SCC and MM were 1.14 (95% CI: 1.03, 1.27), 0.90 (95% CI: 0.70, 1.37) and 1.08 (95% CI: 0.77, 1.50), respectively. The association between radon exposure and BCC was stronger among those with higher socio-economic status and those living in apartments at enrollment.

**Conclusion and Impact:**

Long-term residential radon exposure may contribute to development of basal cell carcinoma of the skin. We cannot exclude confounding from sunlight and cannot conclude on causality, as the relationship was stronger amongst persons living in apartments and non-existent amongst those living in single detached homes.

## Introduction

Radon gas is a radioisotope which has a 3.8 day half-life. Radon-222 gas arises from the radioactive decay of radium-226, present throughout the earth’s crust and in many building materials. The main source of radon in buildings is gas released from the earth underneath and entering the house through cracks in the foundation. Radon gas builds up indoors and main predictors of radon concentrations in Danish buildings include type of soil, basement or not, ventilation rates and type of house (apartment or single-detached home). Radon represents about half of the dose of ionizing radiation received by the Danish population equal to an average of 2 mSv per year, medical diagnostic procedures represent about one fourth, i.e. 1 mSv per year, and the last fourth is shared by various sources such as cosmic radiation and food [1]. The airways and lungs are the primary target organs followed by the skin [2]. Exposure to radon and progeny such as the alpha emitters polonium-218 and polonium-214 [3], has been classified as a human carcinogen [4] and evidence of a link between radon and risk for lung cancer come both from studies of miners exposed to relatively high concentrations and from studies of the general population exposured to radon in the home [5].

Skin cancer, including malignant melanoma (MM) and non-melanoma skin cancer (NMSC), is by far the most common form of cancer in the world, including Denmark [6,7] with exposure to ultraviolet (UV) radiation in sunlight as the key risk factor [8]. Radon and especially its decay products are attracted to water molecules and some atmospheric particles, thus prolonged irradiation of the skin is theoretically possible as these resulting aerosols could adhere to the skin via electrostatic attraction, even after the high personal exposure ceases with possible carcinogenic effects on skin not protected by clothing [9]. Based on a theoretical dosimetry model Eatough and Henshaw concluded that average radon levels of 20 Bq/m^3^ in the United Kingdom could be responsible for 1%-10% of NMSC [10,11]. A more recent estimation based on more justifiable weighting of data sources, latency period and relative biological effectiveness of radiation concluded that the attributable risk of radon exposure for NMSC was 0.5% to 5% at 20 Bq/m^3^ provided that cancer can be initiated in the basal layer of the skin [12]. That review called for case-control or cohort studies to address the issue authoritatively. The most recent review highlighted that the epidemiological evidence relating to risks other than lung cancer was sparse and only evidence of miners exposed to high levels of radon was available for NMSC and MM. That review concluded that there is no epidemiological evidence suggesting that radon exposure contributes directly to excess disease other than lung cancer for which there appeared no lower threshold [13]. Despite these conclusions three British studies with ecological design have shown associations between radon exposure and NMSC [14–16]. However, a key limitation inherent to those studies is the potential for ecological fallacy as associations at the aggregate level do not necessarily reflect associations in individuals. Nevertheless, a cohort study in Iceland showed increased risk of BCC among residents of high-temperature geothermal areas where exposure through water can include radon [17].

We sought to confirm or refute these results using a large prospective cohort design with information on individual radon exposure assessed at the residence by a well-validated model and potential confounders collected at enrollment. We investigate the association between long-term exposure to residential radon and the risk for MM and NMSC (including basal cell carcinoma (BCC) and squamous cell carcinoma (SCC) in Denmark.

## Methods

### The Diet, Cancer and Health Cohort

The present study was based on the prospective Diet, Cancer and Health cohort consisting of 57,053 participants, aged 50 to 64 years, enrolled in 1993–1997 [18]. The participants had to be born in Denmark, live in the Copenhagen or Aarhus areas at the time of enrollment, and be without a cancer diagnosis registered in the Danish Cancer Registry [18]. The baseline examination included a self-administered, interviewer checked, questionnaire on items related to health, lifestyle (including participation in sports, cycling, gardening, walking in leisure time as well as the degree of nevi and freckles on arms) and education [18–20]. The self-reported responses on degree of nevi and freckles on arms at baseline were prompted using pictures that reflected “few” (one or two), “moderate” (more than one or two but in patches) and “high” (all over).

The study was approved by the Scientific Ethics Committee for Copenhagen and Frederiksberg and The Danish Data Protection Agency and written informed consent was obtained from all participants prior to enrollment.

Since establishment of the Danish Civil Registration System (CRS) in 1968 [21], all citizens of Denmark have been given a unique personal identification number, which allows accurate linkage between registers. We used the CRS to obtain information on date of death, emigration or disappearance of the cohort members and information on past and present residential addresses.

### Incident skin cancer cases

Retrieval of all incident cases of MM and NMSC in the “Diet, Cancer and Health” cohort diagnosed during the study period (1993–2011) was carried as follows:

*NMSC*: Each cohort member was linked to an already established NMSC database [22]. This database contains all incident BCC and SCC cases diagnosed in Denmark in the period 1978–2011. The NMSC database was formed by including the first recorded incident BCC or SCC case from either the Danish Cancer Registry or the Danish Registry of Pathology. Both registries have recorded histological types of NMSC separately since 1978. We used the established NMSC database in order to obtain the most precise NMSC incidence numbers for the cohort, as the registration of NMSC in both the Danish Cancer Registry and the Danish Registry of Pathology is known to be incomplete with an estimated registration of 76% [22]. Data on incident cases with origin in the Danish Cancer Registry are classified according to the International Classification of Diseases tenth edition (ICD-10) code C44 and the International Classification of Disease of Oncology third edition (ICD-O-3) as BCC tumors (morphology code M-809) or SCC tumors (morphology code M-807). Data that originated from the Danish Registry of Pathology were classified based on the Danish SNOMED classification topography code T01, T02 and relevant morphology codes. All incident NMSC cases were classified either as BCC tumors (morphology code M-809) or SCC tumors (morphology code M-807). From both registries, only invasive cancers were included (i.e., containing ‘‘3” as the last digit in the morphology code).

*MM*: Incident MM cases were identified by linking each cohort member to the Danish Cancer Registry, which provides accurate and virtually complete nationwide ascertainment of cancers since 1943 [23] and cases were classified according to the International Classification of Diseases tenth edition (ICD-10) code C43.

Each cohort member was followed from date of entry (the first study centre visit) until diagnosis of BCC, SCC, MM or other malignancy, date of death, date of emigration, or end of follow-up on 31 December 2011, whichever occurred first.

### Residential histories

Using the unique personal identification number of the cohort members, we traced residential histories in CRS between 1971 and 2011, thus including over 40 years of address history dating back to when these cohort members were in their 20 to 40’s. We noted the dates of moving in and leaving each address, and linked the addresses to the Danish address database to obtain geographical coordinates (denoted in the following as ‘geocodes’).

### Residential radon exposure assessment

Residential radon concentrations at each address of all participants were predicted with a validated regression model [24]. The model uses nine explanatory variables, including geographic location, geology (soil types) and dwelling characteristics including type of house, floor level, total number of floors, the fraction of inhabitable space in top floor, basement and building materials. All explanatory variables are available from central Danish databases. Model predictions were corrected for seasonal variation. The model and its validation have been described in detail elsewhere [24] and applied in four previous studies of associations between radon and risk of cancer [25–28]

Time-weighted average radon exposure was calculated as the estimated residential radon concentration multiplied by time lived at each address, summed for all addresses lived at during the study period and divided by total observation time, with the unit becquerel per cubic meter.

If radon could not be calculated because of failed geocoding of an address, we imputed the concentration calculated at the preceding address. If the concentration was missing for the first address, we imputed the value at the subsequent address. We included only participants for which we imputed radon for less than 20% of time from 1 January 1971 until diagnosis or censoring.

Sensitivity analyses were carried out to evaluate the effect of imputation, in which we evaluated the effect on reported estimates when considering individuals with complete exposure assessment (no imputation).

### Ultra-violet (UV) radiation exposure from sunlight

UV radiation exposure at each address in the observation period was estimated using data on mean daily duration of bright sunshine obtained from the Danish Meteorological Institute [29], which provides the baseline of the Danish Climate Projections. Denmark was divided into 8 regions in accordance with these sunshine averages, namely Northern Jutland, Mid- and West Jutland, Eastern Jutland, Southern Jutland, Funen, West and Southern Zealand (including Lolland Falster), Copenhagen and Northern Zealand as well as Bornholm. Daily hours of sunshine were based on long-term registration from the DMI aggregated for 1961–1990, giving average daily bright sunshine hours for each month over the 30-year period. The mean yearly-value for each municipality within each of the 8 regions was linked to the municipality code within each of these regions for each included address of cohort participants within the observation period. In average Denmark receives 1495 hours of sunshine each year, with the highest number of hours on Bornholm (1580 hours per year) and the lowest average within mid- and West Jutland (1395 hours per year).

### Statistical methods

The analyses were based on a Cox proportional hazards model with age as the underlying time scale ensuring that risk estimates were based on individuals at exactly the same age [30]. We used left truncation at age of recruitment, so that people were considered at risk from enrollment into the cohort, and right censoring at the age of skin cancer (event), any other cancer, death, emigration, disappearance or end of follow-up on 31 December 2011, whichever came first, separately for BCC, SCC or MM. People diagnosed with cancer prior to enrolment were excluded from the analyses.

Exposure, expressed as time-weighted average residential radon since 1 January 1971 was entered into the statistical skin cancer risk models as a time-dependent variable; thus recalculating exposure for non-censored persons at the time of each censor. The association between radon exposure and BCC, SCC or MM was evaluated with and without adjustment for *a-priori* determined variables. The crude model was adjusted for age (underlying time scale) and sex. In the second model, we further adjusted for individual variables with potential for confounding including skin reaction when exposed to strong sunlight (categorical variable: redness, pain and blistering; redness, pain and peeling; redness, then tan; only tan), degree of freckles on the arms including shoulders and hands (categorical variable: none, few, moderate, or high), degree of nevi on the arms including shoulders and hands (categorical variable: none, few, moderate, or high), body mass index (BMI, linear variable), length of schooling (< 8, 8–10 and > 10 years), socioeconomic status (SES) of baseline municipalities or district for Copenhagen municipality (in total 10 districts) in four groups (low, medium low, medium high and high SES) based on municipality/district information regarding education, work market affiliation and income. Several of these variables have been significantly associated with high-risk tanning behaviour [31–33]. We also adjusted the second model for leisure time physical activities including sports, cycling, walking and gardening (indicator: yes/no) which may be relevant outdoor activities pertinent to sun exposure as well as outdoor occupation for a minimum of 1 year within farming, mining, quarrying, roofing or asphalt road work (indicator yes/no), as higher rates of skin cancer have been reported amongst persons with outdoor occupations [34]. Furthermore, we adjusted model 2 for mean daily hours of bright sunshine at the level of municipality of each residence lived in throughout the observation period.

We formed four intervals for exposure to residential radon using the 25^th^, 50^th^ and 75^th^ percentiles for the time-weighted average radon since 1971 until censoring for all participants as the cut-off points and estimated the IRR for the higher exposure quartiles compared with the lowest exposure quartile separately for each of the sub types of skin cancer. IRRs were also estimated as linear trends in radon concentrations. The assumption of linearity for residential radon in relation to skin cancer sub-types was evaluated graphically using linear splines with boundaries placed at the nine deciles among all participants as well as by a numerical likelihood ratio test statistic to compare the model assuming linearity with the linear spline model. This assumption was valid for all skin sub-types (P > 0.13).

Modification of the association between radon exposure and skin cancer types by sex, school attendance, area level socio-economic status and housing (apartment or single detached home at time of enrolment) was evaluated by introducing interaction terms into the adjusted model and using the Wald’s test. We investigated a possible interaction with housing because both the radon concentrations and the contrasts are much higher in single-detached homes than in apartments; further, the model used to predict radon concentrations in single-detached homes was more advanced than that for apartments [24]. Thus, we would expect a possible association between residential radon and risk for skin cancer to present most clearly in the subgroup of children living in single-detached homes.

The results are expressed as IRRs with two-sided 95% confidence intervals (CIs) on the basis of the Wald test statistic for regression parameters in SAS (version 9.3; SAS Institute, Cary, NC), whereas exposure-response curves with 95% confidence limits were visualized using a restricted cubic spline in R (library Survival and Design, version 2.13.1) [35].

## Results

Among the 57,053 cohort members, 740 were excluded due to a cancer diagnosis (including NMSC) before enrollment; 1,418 because of missing data in potential confounders, and 3,440 because radon exposure was assessed for less than 80% of the time from 1 January 1971 until diagnosis or censoring. The remaining 51,445 cohort participants were followed up for skin cancer for an average of 13.6 years and we identified 3,243 (6.3% cases of BCC), 317 (0.62% cases of SCC) and 329 (0.64% of cases of MM). Excluded participants did not differ from the rest of the cohort with respect to age, education levels, area-level socio-economic status etc. (results not shown).

The median age at recruitment was 56.3 years and skin cancer cases were slightly older. Women represented 52.6% of the study population and sex distribution was similar for the BCC-, MM- cases and cohort participants; whilst a lower proportion of participants were women amongst the SCC cases. Median BMI was 25.6 kg/m^2^ and this was similar among cases and cohort participants. A greater proportion of skin cancer cases had sunlight sensitive skin reacting with redness and pain followed by either blistering or peeling when exposed to strong sunlight; and had a moderate to high degree of freckles or nevi on the arms including shoulders and hands when compared to the whole cohort. The proportion of participants with high to very high municipal-area level SES and long school attendance was similar among SCC, MM cases and cohort members; whilst BCC cases tended to have a slightly higher proportion of participants with very high SES and long school attendance than the rest of the cohort. The majority of subjects participated in gardening or some form of leisure time physical activity: 73.9% did gardening, 53.9% participated in sports, 67.9% cycled and 92.7% walked. The proportion of participants that participated in leisure time physical activity or gardening was similar among SCC, MM cases and cohort members; whilst a higher proportion of BCC cases tended to participate in these activities. Only 10.5% of the participants were employed within an outdoor occupation for at least 1 year, this proportion was slightly higher amongst SCC cases and slightly lower for BCC cases, whilst the proportion of MM cases with employment in outdoor occupation was similar. The median predicted residential radon concentration was 38.3 Bq/m^3^ among cohort participants and levels were higher for skin cancer cases (Table 1).

**Table 1:** Characteristics of all study participants, basal cell carcinoma (BCC), squamous cell carcinoma (SCC) and malignant melanoma (MM).

Characteristic	Cohort	BCC	SCC	MM
Number of participants (%)^a^	51 445 (100)	3 243 (6.30)	317 (0.62)	329 (0.64)
Age at enrolment (years)				
Median	56.3	57.0	59.3	56.7
5^th^– 95^th^ percentile	50.7–64.2	50.8–64.3	51.2–64.7)	50.8–64.2
Number of women (%)	27 050 (52.6)	1 680 (51.8)	127 (40.1)	156 (47.2)
BMI (kg/m^2^)				
Median	25.6	25.1	25.1	25.6
5^th^– 95^th^ percentile	20.4–33.3	20.2–32.1	20.3–32.8	20.3–34.3
Skin reaction to strong sunlight exposure (%)				
Only tan	10 940 (21.3)	522 (16.1)	45 (14.2)	41 (12.4)
Redness, then tan	29 298 (57.0)	1 907 (58.8)	190 (59.9)	196 (59.6)
Redness, pain and peeling	7 943 (15.4)	619 (19.1)	59 (18.6)	71 (21.6)
Redness, pain and blistering	3 264 (6.3)	195 (6.0)	23 (7.3)	21 (6.4)
Freckles (%)				
None	17 930 (34.8)	861 (26.6)	90 (28.4)	71 (21.6)
Few	17 845 (34.7)	1 161 (35.8)	100 (31.6)	97 (29.5)
Moderate	11 936 (23.2)	913 (28.1)	93 (29.3)	112 (34.0)
High	3 734 (7.3)	308 (9.5)	34 (10.7)	49 (14.9)
Nevi (%)				
None	18 901 (36.7)	1 034 (31.9)	138 (43.5)	90 (27.4)
Few	24 595 (47.8)	1 617 (49.9)	133 (42.0)	142 (43.2)
Moderate	7 041 (13.7)	511 (15.7)	39 (12.3)	88 (26.7)
High	908 (1.8)	81 (2.5)	7 (2.2)	9 (2.7)
School attendance (%)				
< 8 years	16 953 (33.0)	910 (28.1)	111 (35.0)	93 (28.3)
8–10 years	23 825 (46.3)	1 560 (48.1)	141 (44.5)	165 (50.1)
>10 years	10 667 (20.7)	773 (23.8)	65 (20.5)	71 (21.6)
Area-level socio-economic status (%)				
Low	7 643 (14.8)	435 (13.4)	43 (13.6)	38 (11.6)
Medium	23 534 (45.8)	1 403 (43.3)	142 (44.8)	163 (49.5)
High	9 835 (19.1)	622 (19.2)	62 (19.5)	59 (17.9)
Very High	10 433 (20.3)	783 (24.1)	70 (22.1)	69 (21.0)
Participation in physical activity in leisure				
Gardening (yes) (%)	37 433 (72.8)	2 482 (76.5)	239 (75.4)	239 (75.4)
Leisure sports (yes) (%)	27 724 (53.9)	1 962 (60.5)	175 (55.2)	175 (55.2)
Cycling (yes) (%)	34 928 (67.9)	2 283 (70.4)	215 (67.8)	215 (67.8)
Walking (yes) (%)	47 706 (92.7)	3 056 (94.2)	293 (92.4)	293 (92.4)
Employment in outdoor occupation (yes) (%)	5 400 (10.5)	290 (8.9)	39 (12.3)	34 (10.3)
Living in single detached home at enrolment (%)	31 115 (60.5)	1 952 (60.2)	191 (60.3)	206 (62.6)
Radon at the address^b^ (Bq/m^3^)				
Median	38.3	42.2	38.8	43.1
5^th^– 95^th^ percentile	9.0–99.5	9.1–102.5	9.0–102.9	9.4–100.5

^a^Row percentages. All other percentages are column percentages.

^b^Time-weighted average radon at the residencies for the period 1 January 1971, to censoring date.

Overall the adjusted IRR per 100 Bq/m^3^ increment increase in residential radon levels for BCC, SCC and MM was 1.14 (95% CI: 1.03, 1.27), 0.90 (95% CI: 0.70, 1.37) and 1.08 (95% CI: 0.77, 1.50), respectively. There was no clear exposure-dependence over the four radon exposure quartiles (quartile cut-offs: < 16.4; 16.4–38.3; 38.3–65.6 and > 65.6 Bq/m^3^) with risk estimates for BCC and SCC being highest for the third quartile and for MM the risk estimate decreased slightly through the quartiles although with wide confidence intervals (Table 2). We found a significant modifying effect of area-level SES on the association between radon exposure and risk of BCC with a stronger association among persons from areas with higher socio-economic status (IRR: 1.31 (95% CI: 1.15, 1.48)) when compared to those from areas with lower economic status (IRR: 1.09 (95% CI: 0.98, 1.22)). Residents living in apartments at recruitment also had significantly stronger associations between radon exposure and risk of BCC (IRR: 1.86 (95% CI: 1.54, 2.26)) when compared to those living in single-detached homes (IRR: 1.05 (95% CI: 0.91, 1.20)). When considering a sub-set of participants based on those that had consistently lived in either a single-detached house (13,049 participants/100 BCC cases) or those consistently living in an apartment throughout follow-up (9,685 participants/606 cases) we found a similar pattern with a stronger association among persons living solely in apartments (IRR: 3.24 (95% CI: 1.14, 9.20)) when compared to those living solely in single-detached homes (IRR: 0.84 (95% CI: 0.68, 1.05)). Finally, the modifying effects of gender on the association between radon exposure and risk of BCC were borderline significant and risk was higher amongst women than amongst men (Table 3), whilst there were no modifying effects of gender on the association between radon exposure and risk of SCC or MM (results not shown). When restricting the study population to those with estimation of radon during the whole time period (without imputation) we found slightly stronger associations for basal cell carcinoma (IRR: 1.17; 95% CI: 1.04–1.33) and malignant melanoma (1.18; 0.81–1.73) but an identical IRR for squamous cell carcinoma (0.90; 0.81–1.73) per 100 Bq/m^3^.

**Table 2:** Association between time-weighted average radon exposure^#^ and basal cell carcinoma, squamous cell carcinoma and malignant melanoma among 51,445 Diet Cancer Health cohort participants.

	Cases,n	Incidence rate ratios (95% CI)	
		Model 1^a^	Model 2^a^ ^,^ ^b^
Basal cell carcinoma			
< 16.4 Bq/m^3^	747	1.00	1.00
(16.4–38.3) Bq/m^3^	746	1.15 (1.05, 1.25)	1.07 (0.98, 1.17)
(38.3–65.6) Bq/m^3^	872	1.34 (1.23, 1.46)	1.20 (1.10, 1.31)
> 65.6	878	1.28 (1.18, 1.40)	1.15 (1.05, 1.26)
*Linear trend per 100 Bq/m* ^*3*^	*3243*	*1*.*32 (1*.*20*, *1*.*44)*	*1*.*14 (1*.*03*, *1*.*27)*
Squamous cell carcinoma			
< 16.4 Bq/m^3^	83	1.00	1.00
(16.4–38.3) Bq/m^3^	73	0.90 (0.69, 1.18)	0.89 (0.65, 1.15)
(38.3–65.6) Bq/m^3^	76	1.22 (0.87, 1.45)	1.00 (0.76, 1.33)
> 65.6 Bq/m^3^	85	1.06 (0.82, 1.38)	0.95 (0.72, 1.27)
*Linear trend per 100 Bq/m* ^*3*^	*317*	*1*.*12 (0*.*83*, *1*.*50)*	*0*.*90 (0*.*70*, *1*.*37)*
Malignant melanoma			
< 16.4 Bq/m^3^	62	1.00	1.00
(16.4–38.3) Bq/m^3^	87	1.43 (1.09, 1.87)	1.37 (1.04, 1.82)
(38.3–65.6) Bq/m^3^	85	1.36 (1.04, 1.80)	1.27 (0.94, 1.71)
> 65.6 Bq/m^3^	95	1.37 (1.04, 1.81)	1.24 (0.92, 1.69)
*Linear trend per 100 Bq/m* ^*3*^	*329*	*1*.*24 (1*.*09*, *1*.*58)*	*1*.*08 (0*.*77*, *1*.*50)*

^a^Adjusted for age (underlying time scale) and sex.

^b^Adjusted for skin reaction to sunlight, degree of freckles, degree of nevi, BMI, school attendance, area-level socio-economic status, leisure time physical activities including sports, cycling, walking and gardening as well as outdoor occupation (farming, mining, quarrying, roofing or asphalt road work) and mean daily hours of bright sunshine at the level of municipality of each residence.

Due to exclusion of cohort members with missing value in any covariate, the number of persons is identical in the crude and the adjusted analyses.

^#^Radon exposure was entered as a continuous variable in all models as the time-weighted average at residences from 1 January 1971 until censoring.

**Table 3:** Modifications of associations between time-weighted average residential radon exposure^a^ (per 100 Bq/m^3^) and basal cell carcinoma cases (n = 3243) among the 51,445 DCH cohort participants.

Potential effect modifier (at enrolment)	Incidence Rate Ratio (95% CI)^b^ ^,^ ^c^ ^,^ ^d^	P^f^
Sex		
Males	1.04 (0.90, 1.20)	0.07
Females	1.24 (1.08, 1.42)	
School attendance (years)		
< 8	1.20 (1.00, 1.43)	0.53
≥ 8	1.12 (0.99, 1.26)	
Area-level socio-economic status		
Low/medium	1.09 (0.98, 1.22)	0.01
High/very high	1.31 (1.15, 1.48)	
House index		
Single-detached home	1.05 (0.91; 1.20)	0.01
Apartment	1.86 (1.54; 2.26)	

^a^Radon exposure was entered as a continuous variable in all models as the time-weighted average residential concentration (Bq/m^3^) from 1 January 1971 until censoring.

^b^We adjusted for age (underlying time scale), sex, skin reaction to sunlight, degree of freckles, degree of nevi, BMI, school attendance, area-level socio-economic status, physical sports (sport, cycling and walking) and gardening, as well as outdoor occupation (farming, mining, quarrying, roofing or asphalt road work for at least 1 year) and mean daily hours of bright sunshine at the municipality level of each residence lived in throughout the observation period.

^c^No adjustment for the modification variable.

^d^IRR expressed per 100 Bq/m^3^ radon exposure.

^f^Test of the null hypothesis that the linear trends are identical, for Wald test for interaction.

**Fig 1:**
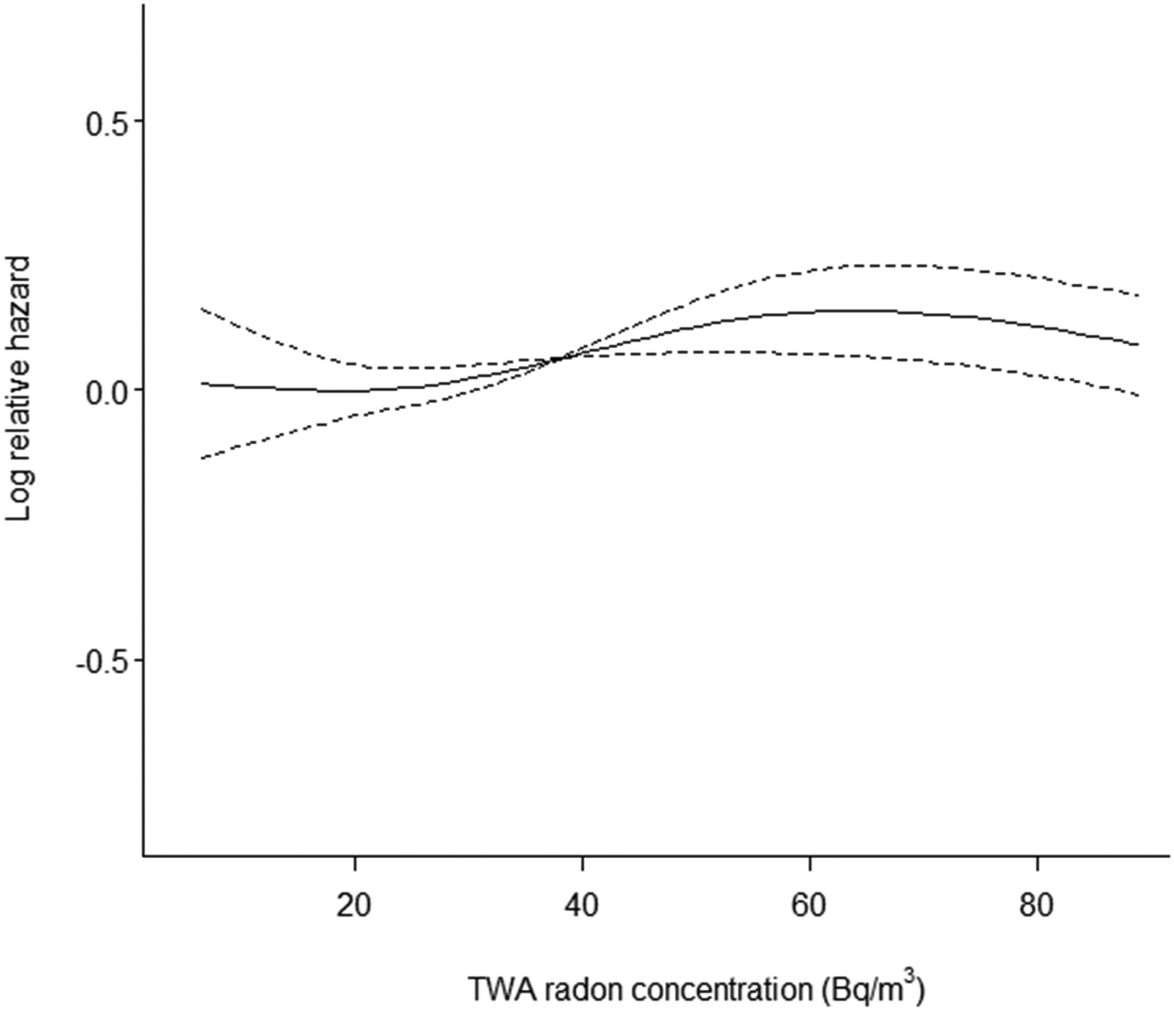
Spline functions (filled lines; 95% CIs indicated by dashed lines) between BCC and average residential radon concentration (Bq/m^3^) at the residencies from 1971 onwards, based on the fully adjusted model 2 and cohort participants with exposure between 5^th^ and 95^th^ percentile.

Fig 1 shows the exposure-response curve of BCC based on the fully adjusted model 2. The IRR for BCC increased with exposure up to around 60 Bq/m^3^ and then appeared to level off although with wide confidence intervals and not refuting a linear relationship. The associations observed for SCC and MM were not convincing with respect to exposure-response relationships (Figs not shown).

## Discussion

We found a significant association between long-term exposure to residential radon and BCC in a general Danish population.

This prospective cohort study investigated long-term exposure to residential radon, estimated at each individual address in association with incidence of NMSC (BCC and SCC) and MM assessed from nationwide registers, where information on a wide range of potential confounding factors was collected at enrollment without potential for recall bias. Residential radon is responsible for exposure to ionizing radiation corresponding to a 2 mSv dose per year, representing half of the total dose received by the Danish population at large, and ionizing radiation has been implicated as a risk factor for the development of BCC [36,37]. Radon progeny deposited on skin especially areas not protected by clothing could thus contribute to BCC usually developing in those areas of the body which are also exposed to UV radiation in sunlight. Although alpha particles from radon progeny out-plated on the skin have limited penetration to hair follicles and/or deep dermis suggested by earlier animal studies to be important for skin cancer development [38], the estimated attributable risk of skin cancer was 0.5% to 5% at indoor radon levels of 20 Bq/m^3^ assuming that the basal layer of the epidermis is the target [12]. The IRR for BCC of 1.14 per 100 Bq/m^3^ found in the present study corresponds to an attributable risk of approximately 3% given a mean population exposure of 20 Bq/m^3^, which is in line with the findings of Charles (2007b). The median exposure in our cohort was 38 Bq/m^3^ and if the association is causal this would imply a clinically relevant risk at population level, although of much smaller magnitude than that conveyed by exposure to sunlight.

Only studies with ecological design have previously investigated the association between radon exposure at residential level and the risk of skin cancer in a general population finding significant associations with NMSC [14–16]. One of these studies reported a significant association between residential radon and SCC but not BCC and MM [15]. We do not corroborate that result in the present study, but as the authors themselves outline, their study had inherent limitations regarding ecological fallacy and they cannot be certain that the association they report at the aggregate level is also relevant at the individual level.

We found statistically significant associations for BCC and not for SCC or MM in relation to radon exposure which may be due to the much higher number of BCC cases improving statistical power. The association was stronger among women, persons living in apartments at enrolment and subjects from areas with high socio-economic status; the latter two with significant interaction terms. It seems counter-intuitive that an association was detected among persons living in apartments at enrolment but not single-detached homes. When considering the effect of housing status of the cohort participants from enrolment onwards we found a similar effect amongst participants whom had only lived in an apartment throughout this period compared to those whom had only lived in single detached homes. This may indicate that the association we find is non-causal since radon exposure and exposure variations among apartments are believed to be smaller and less well modelled than radon exposure variations among single-family houses [24]

If the apparent association between radon and BCC is caused by confounding by sun exposure, this might cause the observed pattern since high-risk sun-tanning behavior is more frequent among women providing higher exposure to sunlight and a wider exposure gradient allowing for more effect as confounder. This could also relate to individuals of higher socioeconomic status and among those living in larger cities among whom high-risk sun-tanning behavior is more frequent [31].

The present study includes individual historical estimates of radon exposure in more than 180,000 homes, since 1971 when the cohort members were in their 20’s to 40’s, providing much more detailed exposure information than previous studies of radon and skin cancer [14–16]. Residential level of radon has been shown to be a reasonably good proxy of personal exposure with r^2^-values of 0.52 to 0.85 [39,40]. The recently developed regression model for exposure assessment has been applied in four previous epidemiological studies [25–28] and successfully validated against independent radon measurements [24]. Nevertheless, modeling of radon levels is inevitably associated with some uncertainty but actual measurements in the homes could only assess current levels incurring extreme costs and most likely low participation rate. Imputation methods may introduce uncertainty; however analyses based on participants with complete exposure assessment (no imputation) showed similar results.

Exposure of cohort members before 1971 could not be estimated, as residential histories before that date were unknown. Therefore, we were unable to assess early-life radon exposure which is an important limitation as early life environmental exposures might be most significant for cancer risk. Also our estimation of radon exposure is based on residential addresses of cohort members and we cannot account for radon at temporary addresses abroad or in indoor work environments.

Finally, a key risk factor for skin cancer is UV radiation from sunlight. The limited variation in solar irradiance across the relatively small country of Denmark as well as the lower level of UV radiation we experience compared with other countries that are located closer to the equator [41], means that UV exposure variation is largely dictated by behavior. High risk tanning behaviour in Denmark has been associated with female gender, younger age, shorter education, skin type 3 or 4 (based on sun exposure, nevi and freckling), higher socio-economic status and living in larger cities [31]; these variables were adjusted for in this study. We further adjusted for leisure time physical activities, outdoor occupation and daily hours of bright sunshine at the level of municipality of each residence, but since we have no direct information on exposure to sunlight, the association between radon and BCC detected in the present study might be due to residual confounding from sunlight exposure.

The association we find between residential radon and BCC may not be causal. But the potential implications of a causal association are important and would emphasize the need to reduce indoor radon concentrations, e.g. by increasing ventilation rates and sealing foundations so residents are protected from radon.

## Conclusion

This large cohort study with detailed individual exposure assessment and control of several potential confounders showed that long-term residential exposure may contribute to development of basal cell carcinoma of the skin. We cannot exclude confounding from sunlight exposure, nor can we conclude on causality as the relationship was stronger amongst persons living in apartments and non-existent amongst those living in single detached homes.
